# Therapeutic Challenges in Metastatic Myxofibrosarcoma: A Case-Based Review

**DOI:** 10.7759/cureus.93954

**Published:** 2025-10-06

**Authors:** Trie Arni Djunadi, Angela Grigos, Svetoslav Bardarov, Shamim Salman

**Affiliations:** 1 Internal Medicine, Richmond University Medical Center, Staten Island, USA; 2 Hematology and Oncology, Richmond University Medical Center, Staten Island, USA; 3 Pathology and Laboratory Medicine, Richmond University Medical Center, Staten Island, USA

**Keywords:** myxofibrosarcoma, oncology, pulmonary metastases, sarcoma, treatment

## Abstract

Myxofibrosarcoma (MFS) is a histologically distinct and aggressive subtype of soft tissue sarcoma, most often affecting elderly individuals and commonly presenting in the extremities. Despite standard treatment modalities, including surgical resection, radiotherapy, and chemotherapy, MFS is notable for its high recurrence and metastatic potential.

We present the case of a 64-year-old woman with stage IIIA MFS of the right thigh and progressive pulmonary metastases, despite undergoing radiotherapy, surgery, and systemic chemotherapy. Her disease course was complicated by genetic alterations, including NF1 and ATM mutations, suggesting potential responsiveness to targeted therapies such as MEK and PARP inhibitors. Histological and immunohistochemical findings confirmed a fibrohistiocytic origin, consistent with metastatic MFS.

This case highlights the diagnostic and therapeutic challenges associated with MFS, emphasizing the need for improved molecular characterization and novel therapeutic strategies. While current treatments provide limited durable control, emerging insights into the tumor’s genetic and immune landscape offer hope for more effective, personalized approaches.

## Introduction

Myxofibrosarcoma (MFS) is among the most prevalent types of soft tissue sarcoma (STS) in adults, comprising approximately 1% of all adult cancers. Originally considered a myxoid variant of malignant fibrous histiocytoma, MFS was reclassified as a distinct histological subtype in 2002 [1,2]. This aggressive sarcoma is characterized by malignant fibroblastic cells within a myxoid stroma, marked cellular pleomorphism, and a distinctive pattern of curvilinear blood vessels. MFS most frequently affects the extremities of elderly patients and has the highest recurrence rate of any STS, with reported rates between 20 and 60% [1,3,4]. The lack of specific immunohistochemical markers further complicates accurate diagnosis and therapeutic decision-making. Current management strategies focus on surgical resection, radiotherapy, and chemotherapy, yet recurrence and metastasis remain significant clinical challenges [5,6]. Molecular profiling has emerged as a valuable tool for identifying actionable mutations, informing targeted therapies, and guiding personalized treatment approaches.

In this case report, we present a patient with stage IIIA MFS who has developed progressive metastatic recurrence despite multiple treatment interventions. We review her clinical course, explore current treatment options, and discuss potential future therapeutic approaches that may improve outcomes in similar cases.

## Case presentation

We present the case of a 64-year-old female with a complex medical history, including diabetes mellitus (DM), hypertension (HTN), hyperlipidemia (HLD), chronic smoking, right thigh MFS with pulmonary metastasis, asthma, and a non-toxic multinodular goiter. She presented to the Emergency Department (ED) with worsening shortness of breath and a productive cough with white sputum persisting for the past three weeks. She also reported a sore throat without nasal congestion, as well as back pain likely related to persistent coughing.

On examination, the patient was alert, oriented, and not in acute respiratory distress, though she exhibited diffuse bilateral wheezing. She was an active smoker. Laboratory workup revealed a hemoglobin level of 8.6 g/dL. Chest computed tomography (CT) demonstrated multiple bilateral pulmonary nodules with peripheral consolidations, consistent with both metastatic disease and possible infection. Dependent fluid within the trachea and bronchi, along with bibasilar consolidative opacities, suggested aspiration pneumonia. Given her history of MFS, the oncology service was consulted to evaluate for sarcoma progression, metastatic disease, or chemotherapy-related toxicity.

**Figure 1:**
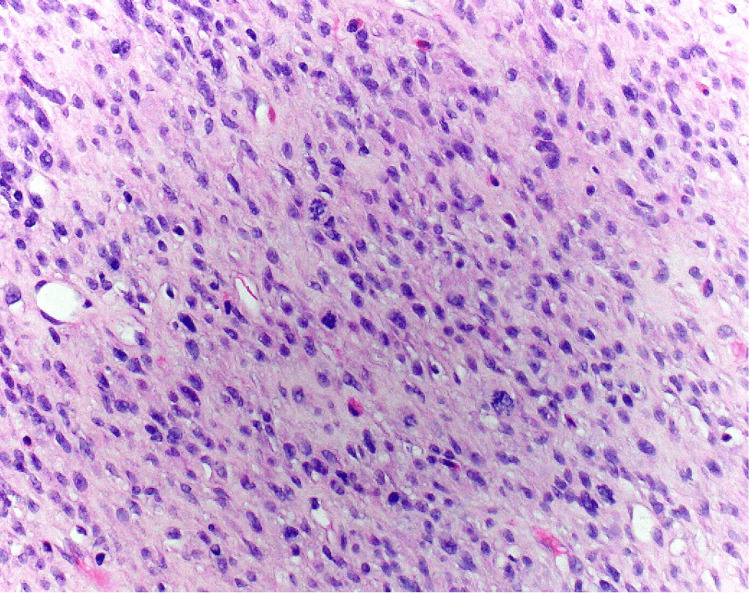
On histology examination, the biopsy showed high-grade sarcoma with immunostaining positive for CD163, Factor XIIIa, and FLI-1, and a Ki-67 proliferation index >20%.

The patient was first evaluated in June 2023 for an enlarging right thigh mass that had been present for approximately one year and was initially attributed to trauma. Magnetic resonance imaging (MRI) revealed a heterogeneous subcutaneous soft tissue mass concerning for sarcoma. Biopsy confirmed a high-grade sarcoma (Figure 1), with immunostaining positive for CD163, Factor XIIIa, and FLI-1, and a Ki-67 proliferation index >20%. Stains were negative for AE1/3, HHV-8, DOG1, CD34, S100, CD56, SMA, and Desmin, consistent with a fibrohistiocytic origin and most suggestive of dermatofibrosarcoma protuberans (DFSP). Molecular profiling identified an NF1 V141fs mutation, potentially targetable with selumetinib, and subsequent liquid biopsy revealed an ATM L516fs mutation, suggesting potential sensitivity to PARP inhibitors such as olaparib or talazoparib. A staging CT in July 2023 revealed no pulmonary involvement.

In October 2023, she underwent surgical resection of the right thigh mass. Pathology confirmed high-grade MFS with 10% necrosis, without lymph node involvement or metastasis. She subsequently completed 30 sessions of adjuvant radiotherapy between October and November 2023, achieving remission.

**Figure 2:**
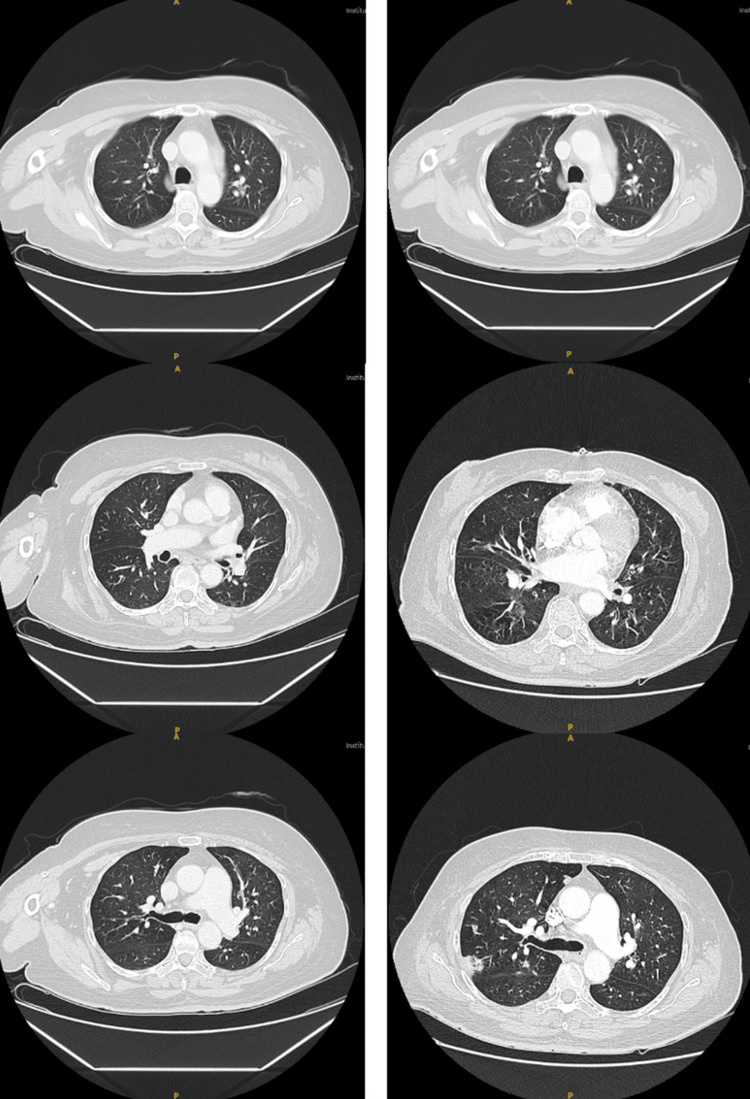
CT images of the chest. The scan obtained in June 2023 (left) shows no evidence of pulmonary lesions, while the follow-up scan (right) demonstrates metastatic nodules in the lungs.

Surveillance CT chest in April 2024 demonstrated new right lower lobe opacities suspicious for pulmonary metastases (Figure 2). A lung biopsy in May 2024 confirmed metastatic MFS. Systemic chemotherapy with doxorubicin and dexrazoxane was initiated shortly thereafter. She completed two cycles of treatment, with the third planned for the following week, when she presented to the ED.

At present, the patient is classified as Stage IIIA progressive metastatic MFS, unresponsive to prior local therapy. She remains on systemic chemotherapy and was admitted with respiratory distress secondary to community-acquired pneumonia, for which empiric levofloxacin was initiated.

## Discussion

MFS is a subtype of STS predominantly affecting men in their sixth to eighth decades of life. It typically presents as a slow-growing, painless mass, most commonly in the lower extremities, though it can occasionally appear in the upper extremities and, rarely, in the trunk, head, neck, hands, or feet. Macroscopically, superficial MFS lesions often exhibit variably gelatinous or firm nodules, while deep-seated lesions display infiltrative margins. Over time, MFS lesions tend to become more cellular and higher in grade, often due to local recurrence [1,7,8]. While MFS lacks consistent cytogenetic markers, it is characterized by complex structural and numerical chromosomal aberrations, indicating genetic instability that progresses in a multistep manner [7,9]. Intratumoral mutational heterogeneity is common, with mutations often affecting the p53 pathway and clusters of methylation, including hypermethylation. Currently, no specific immunohistochemical markers exist for diagnosing MFS, as it displays complex karyotypes and shares many genetic abnormalities with other STS types [7,10].

The prognosis for MFS is challenging due to its high recurrence rate, reported to range from 20% to 60% within five years. Among these recurrences, 15% to 38% may progress to higher histological grades, with an increased risk of metastasis. The overall incidence of distant metastasis is estimated at 20% to 25% [1,8,11]. Poorer outcomes in MFS have been associated with genetic alterations, such as TP53 mutations and KRAS amplifications, as well as high integrin-α10 expression in high-grade cases [10]. Furthermore, a gene expression signature known as CINSARC has demonstrated potential in predicting metastatic risk in sarcomas. Preoperative MRI is valuable for identifying cases at high risk of local recurrence, especially when features like a high myxoid matrix, marked contrast enhancement, and an infiltrative “tail sign” are observed [12,13].

The primary treatment for localized MFS is surgical resection with histologically confirmed clear margins, often combined with neoadjuvant or adjuvant radiotherapy. Amputation is reserved for extreme cases. The benefit of radiotherapy for local tumor control remains unclear; although some retrospective studies indicate a lower risk of local recurrence in retroperitoneal STS, there is evidence that MFS may exhibit increased radioresistance. Common radiotherapy (RT) protocols for STS with wide or marginal surgical margins include 50 Gy in 25 fractions or 36 Gy in hyperfractionated doses of 1.8 Gy twice daily when combined with chemotherapy. Intralesional margins require higher doses, typically 64-66 Gy in 2 Gy fractions, or 45 Gy with hyperfractionation [1,7]. Chemotherapy is primarily used for palliation in metastatic MFS, with limited effectiveness. First-line treatments for recurrent or metastatic MFS include doxorubicin and ifosfamide, yielding response rates of 20-30%. Dacarbazine may be added, while second-line options such as gemcitabine alone or with docetaxel are utilized due to MFS’s similarity to gemcitabine-sensitive undifferentiated pleomorphic sarcoma (UPS) [1,14]. Targeted therapies, like the tyrosine kinase inhibitor pazopanib, have shown promise for advanced STS (excluding liposarcoma) and are used as palliative options for progressive, unresectable, or metastatic cases, with studies ongoing to explore multi-pathway targeting for enhanced efficacy [15].

PARP inhibitors (olaparib, niraparib, and talazoparib) are most effective in tumors with defective homologous recombination repair (HRR) pathways, including mutations in BRCA1/2, ATM, PALB2, CHEK2, and NF1. Although BRCA mutations are rare in sarcomas, alterations in ATM and NF1 have been reported in myxofibrosarcoma, suggesting a potential therapeutic vulnerability. A recent phase Ib trial involving 41 patients, most with undifferentiated pleomorphic sarcoma, demonstrated that combining PARP inhibition with radiotherapy achieved a median progression-free survival (PFS) of 7.7 months with an acceptable safety profile, highlighting its promise as a strategy for unresectable tumors. While the combination of PARP inhibitors and radiotherapy has been evaluated in various solid tumors, evidence remains limited for myxofibrosarcoma specifically [16].

The MEK inhibitor selumetinib is an approved treatment for neurofibromatosis type 1. A recent unique case report described clinical improvement in a patient with myxofibrosarcoma harboring a RAF1 S259P mutation and CDKN2A/Bloss who was treated with a combination of the MEK inhibitor trametinib and the CDK4/6 inhibitor palbociclib. This finding suggests that MEK inhibition may be effective in myxofibrosarcoma, but likely only in patients with specific genetic mutations. It also highlights that selumetinib itself was not the MEK inhibitor used in this particular case [17].

Despite significant research efforts in recent years, advances in understanding the pathogenesis and development of MFS have yet to translate into substantial improvements in clinical outcomes. Immunogenomic studies suggest that complex-karyotype sarcomas like MFS often have an immune-infiltrated tumor microenvironment (TME), indicating some potential for immunotherapy responsiveness. However, response rates remain low relative to other tumor types, as STS generally exhibits low immunogenicity, leading to an immune-poor TME with limited targetable molecules. Continued research into the molecular and immunological characteristics of MFS, alongside investigations into multi-modal therapies, holds promise for improving the prognosis of this challenging sarcoma subtype [18,19].

## Conclusions

In conclusion, MFS remains a therapeutically challenging soft tissue sarcoma due to its high recurrence rate, metastatic potential, and limited systemic treatment options. Despite advances in surgical and radiotherapeutic management, long-term outcomes remain poor, underscoring the need for novel therapeutic approaches. Molecular alterations in NF1, ATM, and RAF1 suggest potential roles for targeted agents such as MEK or PARP inhibitors in select patients. Although evidence remains limited, these findings support the promise of precision medicine in MFS. Recognition of an immune-infiltrated tumor microenvironment also suggests potential variable responsiveness to immunotherapy. Ongoing research integrating molecular profiling with targeted and immune-based therapies may help optimize outcomes and guide personalized treatment approaches for advanced or metastatic MFS.
